# Supplementary material for: Cathepsin S Upregulation Measured in the Peripheral Blood Mononuclear Cells Prior to Surgery Points to Postoperative Pain Development in Patients with Hip Osteoarthritis
Source: Diagnostics (Basel). 2023 May 15;13(10):1739. doi: 10.3390/diagnostics13101739 (PMC10217262; doi:10.3390/diagnostics13101739)
Supplement: Supplementary file 1 [file diagnostics-13-01739-s001.zip › diagnostics-2348321-supplementary.pdf]

Table S1. Baseline relative expression of cathepsin S, TNFα, IL1β, and COX2 genes, determined by real-time PCR in the blood of end-stage HOA patients who either developed postoperative pain (n=12) or painless subjects (n=19), compared with healthy individuals

Patient #	Cathepsin S	IL-1β	TNFα	COX2
Patients who developed pain six months after surgery (n=12)				
1	77,68425	15,7927	12,1254	10,4975
2	20,44005	4,11678	1,77461	8,55146
3	10,20037	0,75505	3,09962	2,82093
4	3,564838	0,55807	0,3103	1,33883
5	5,41791	5,11678	2,66307	2,45936
6	4,320885	0,46766	2,2239	0,06383
7	3,806725	0,10665	0,93029	0,25701
8	5,69516	0,65807	0,12808	0,39071
9	8,327229	0,85505	3,66307	1,32013
10	134,3185	17,7927	2,0239	5,04191
11	20,44005	0,38174	1,93029	3,68885
12	10,20037	0,36766	0,52808	2,5
Pain-free patients six months after surgery (n=19)				
1	1,106748	0,10665	2,06779	0,41557
2	3,642802	0,55807	1,3129	0,39978
3	37,40213	16,7927	1,17176	0,86344
4	2,59287	0,46766	1,16595	4,09871
5	11,94915	4,8556	14,5758	2,31063
6	8,797226	0,95505	9,90053	2,47937
7	0,740745	0,38174	7,7674	3,4435
8	22,1318	15,7927	5,32029	2,67937
9	5,693543	0,75505	1,51546	2,06333
10	1,682652	0,56766	2,20458	0,48689
11	6,453352	4,11678	1,13564	3,0435
12	1,904387	0,20201	1,11845	7,11707
13	2,495015	0,20665	2,51825	2,30198
14	1,106748	0,45807	5,02029	0,27281
15	3,642802	0,8491	1,91546	2,01063
16	2,59287	0,87298	2,00458	1,43115
17	11,94915	3,11678	1,23564	6,11707
18	8,797226	4,3556	1,31845	3,09871
19	0,740745	1,01996	2,41825	1,83115
